# Protease-activated alpha-2-macroglobulin can inhibit amyloid formation via two distinct mechanisms

**DOI:** 10.1016/j.febslet.2013.01.020

**Published:** 2013-03-01

**Authors:** Amy R. Wyatt, Patrick Constantinescu, Heath Ecroyd, Christopher M. Dobson, Mark R. Wilson, Janet R. Kumita, Justin J. Yerbury

**Affiliations:** a Illawarra Health & Medical Research Institute, School of Biological Sciences, University of Wollongong, Northfields Avenue, Wollongong, NSW 2522, Australia; b Department of Chemistry, University of Cambridge, Lensfield Road, Cambridge CB2 1EW, UK

**Keywords:** α_2_M, α_2_-macroglobulin, LRP, lipoprotein receptor-related protein, trypsin-α_2_M, trypsin-activated α_2_M, (*i*)trypsin-α_2_M, trypsin-activated α_2_M treated with small molecule protease inhibitors, ThT, thioflavin T, α_2_-Macroglobulin, Extracellular chaperone, Amyloid disease, Human lysozyme, Aβ_1–42_

## Abstract

α_2_-Macroglobulin (α_2_M) is an extracellular chaperone that inhibits amorphous and fibrillar protein aggregation. The reaction of α_2_M with proteases results in an ‘activated’ conformation, where the proteases become covalently-linked within the interior of a cage-like structure formed by α_2_M. This study investigates, the effect of activation on the ability of α_2_M to inhibit amyloid formation by Aβ_1–42_ and I59T human lysozyme and shows that protease-activated α_2_M can act via two distinct mechanisms: (i) by trapping proteases that remain able to degrade polypeptide chains and (ii) by a chaperone action that prevents misfolded clients from continuing along the amyloid forming pathway.

**Structured summary of protein interactions:**

**Aβ_1–42_** and **Aβ_1–42_** bind by fluorescence technology (View interaction)**I59T lysozyme** and **I59T lysozyme** bind by light scattering (View interaction)**I59T lysozyme** and **I59T lysozyme** bind by fluorescence technology (View interaction)Alpha-lactalbumin and Alpha-lactalbumin bind by fluorescence technology (View interaction)**I59T lysozyme** and **I59T lysozyme** bind by electron microscopy (View interaction)**Aβ_1–42_** and **Aβ_1–42_** bind by electron microscopy (View interaction)

## Highlights

► α_2_M is an extracellular chaperone able to inhibit protein aggregation. ► Protease–α_2_M complexes can degrade amyloidogenic substrates or act as a chaperone. ► Activated α_2_M may play an important role in preventing protein deposition in vivo*.*

## 1 Introduction

α_2_-Macroglobulin is a highly abundant glycoprotein present in blood plasma, cerebrospinal fluid and other extracellular fluids. α_2_M is best known for its ability to trap a broad range of proteases within a cage-like quaternary structure via covalent-linkage of the protease to intramolecular thioester bonds on α_2_M [1]. This reaction results in a conformationally altered form commonly known as “activated” or “fast” α_2_M, the latter term relating to enhanced mobility via native gel electrophoresis. Activation of α_2_M results in the exposure of a cryptic receptor recognition site for the low-density lipoprotein receptor-related protein (LRP) [1]. In addition to proteases, small nucleophiles can activate α_2_M by interacting directly with its thioester bonds [2].

Along with protease trapping, many other biological functions have been proposed for α_2_M; including roles in immunomodulation, cancer progression and extracellular proteostasis [3–5]. α_2_M can bind to a range of endogenous disease-associated proteins including the amyloid β peptide (Aβ_1–42_) [6], prion proteins [7] and β_2_-microglobulin [8], which are the main components of deposits found in Alzheimer’s disease (AD), spongiform encephalopathies and dialysis-related amyloidosis, respectively [9]. Moreover, α_2_M is found to be co-localized in vivo with amyloid deposits in AD and the spongiform encephalopathies [7,10]. Recent work has shown that native α_2_M can act as an ATP-independent molecular chaperone by suppressing stress-induced amorphous protein aggregation [5]. The mechanism by which this occurs appears to involve the formation of stable, soluble complexes between α_2_M and the misfolded client proteins [5]. Native α_2_M has also been shown to suppress the fibril formation of a range of amyloidogenic proteins and peptides [11,12]. It has been proposed that α_2_M can protect against pathogenic misfolded proteins by promoting their removal from the extracellular space [6,13,14]. However, trypsin-activated α_2_M (trypsin-α_2_M) is reportedly unable to prevent the amorphous aggregation, in vitro, of some proteins [5]. Nevertheless, after binding to misfolded proteins, α_2_M retains the ability to become activated, and α_2_M-trypsin-misfolded protein complexes are recognized by LRP [5], representing a potential route for the targeted disposal of misfolded proteins in vivo.

Activated α_2_M can protect cells from Aβ toxicity in vitro through specific binding and subsequent LRP mediated uptake and degradation of Aβ_1−40_ [6,10,15]. While it is clear that activated α_2_M can bind to Aβ peptide, its ability to prevent the fibrillar aggregation of amyloid forming peptides or proteins has not been tested. To address this issue, we investigate the effect of activated α_2_M on the fibril formation of the amyloidogenic Aβ_1–42_ peptide and of a non-natural variant of human lysozyme (I59T) that possesses many attributes associated with the natural amyloidogenic variants linked to systemic amyloidosis [16].

## 2 Materials and methods

Chemicals and reagents were purchased from Sigma–Aldrich Ltd. unless otherwise stated.

### 2.1 Proteins and peptides

α_2_M was purified from human plasma by zinc chelate affinity chromatography and size exclusion chromatography (SEC) as previously described [5]. Purified α_2_M was stored at 4 °C (for less than 2 months) and routinely examined prior to use by native polyacrylamide gel electrophoresis (PAGE) to ensure that the preparation had not become partially degraded, activated or cross-linked, modifications that can occur with prolonged storage [4,17,18]. Aβ_1–42_ was purchased from Biopeptide Co. Inc. or Bachem AG. Solutions of Aβ_1–42_ peptide were prepared by a TFA/HFIP dissolution method [19]. The non-natural variant of human lysozyme, I59T, was expressed and purified as previously described [16].

### 2.2 Preparation of activated α_2_M

Trypsin-α_2_M was prepared by incubating α_2_M with a threefold molar excess of bovine trypsin in PBS (pH 7.4, 25 °C, 45 min). The degree of α_2_M activation was assessed by NuPAGE Novex 3–8% Tris–acetate gels with Tris–glycine native running buffer (Life Technologies Ltd.). The reaction was allowed to continue for up to an additional 45 min to ensure completion. Unreacted trypsin was removed by SEC and SDS–PAGE analysis using NuPAGE Novex 4–12% Bis–Tris gels with MES running buffer (Life Technologies Ltd.) confirmed that no cleavage outside the bait region had occurred. To produce enzymatically inactivated trypsin-α_2_M (i.e. (*i*)trypsin-α_2_M), trypsin-α_2_M was incubated (2 h, 25 °C) with excess Complete™ protease inhibitor cocktail (Roche Diagnostics Ltd.) and samples were desalted using Zeba™ desalting columns (Thermo Fisher Scientific). Ammonium chloride (NH_4_Cl) activation was performed by incubating α_2_M with 400 mM NH_4_Cl in PBS (14 h, 25 °C) and subsequently desalting as described.

### 2.3 Thioflavin-T assays

Aβ_1–42_ (5 μM, PBS (pH 7.4), 50 μM ThT) was incubated in a 384 well plate (37 °C, with shaking) using a FLUOstar OPTIMA fluorescence plate reader (BMG Labtech Ltd.) with excitation and emission wavelengths of 440 nm and 480 nm (slit-widths 10 nm). I59T lysozyme (6.8 μM, 0.1 M citrate buffer (pH 5.0), 25 μM ThT) was incubated with stirring at 60 °C in a Cary Eclipse spectrofluorimeter (Agilent Ltd.) and ThT fluorescence intensity was monitored with excitation and emission wavelengths of 440 nm and 480 nm (slit-widths 5 nm). All samples incubated with native α_2_M, trypsin-α_2_M, (*i*)trypsin-α_2_M, or NH_4_Cl-activated α_2_M contained a molar ratio of substrate-to-α_2_M of 10:1, based on the molecular weights of the α_2_M tetramer (720 kDa), the Aβ_1–42_ monomer (4.5 kDa) or the I59T monomer (14.7 kDa). All experiments were performed in triplicate.

### 2.4 SDS–PAGE analysis

At the endpoints of the aggregation assays, aliquots were removed and either centrifuged (10 min, 10 000×*g*) (I59T lysozyme and Aβ_1–42_) or filtered (0.22 μm filter) (I59T lysozyme). For I59T lysozyme, the pellet fractions were rinsed with dH_2_O, centrifuged again (10 min, 10 000×*g*) and then dissolved in 10 μl of 8 M urea solution. The supernatants and solubilised pellets were separated on 4–12% NuPAGE gels under reducing conditions. The gels were stained with Coomassie Brilliant Blue or Sigma ProteoSilver stain kit for I59T and Aβ_1–42_, respectively. Additionally, Aβ_1–42_ labelled with Hilyte™ 488 (AnaSpec) was incubated with 10:1 substrate-to-trypsin-α_2_M (30 min, 25 °C) and centrifuged (10 min, 10 000×*g*). The supernatants were separated on 4–12% NuPAGE gels and visualized using a Typhoon Trio Imager (GE Healthcare Ltd).

### 2.5 Transmission electron microscopy (TEM)

Fibril solutions (5 μl) were applied to carbon-coated nickel grids, stained with 2% (w/v) uranyl acetate, and imaged on a FEI Tecnai G_2_ transmission electron microscope (Multi-Imaging Unit in the Department of Physiology, Development and Neuroscience, University of Cambridge, UK). Images were analyzed using the SIS Megaview II Image Capture system (Olympus).

## 3 Results

**Fig. 1:**
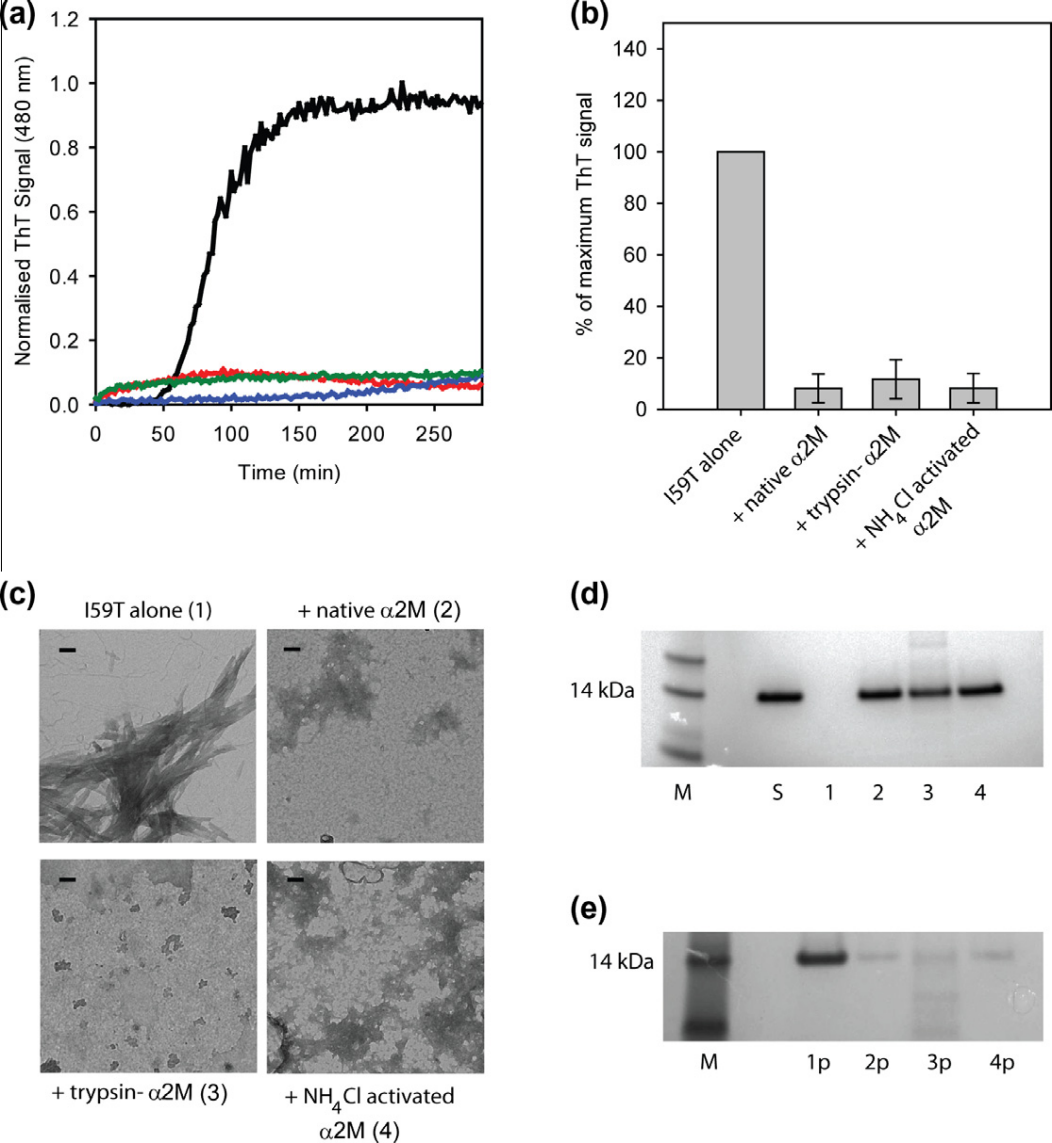
Effects of α_2_M variants on I59T lysozyme fibril formation. (a) In vitro fibril formation of I59T lysozyme incubated alone (black), with native α_2_M (red), with trypsin-α_2_M (blue) or with NH_4_Cl-activated α_2_M (green) using α_2_M-to-lysozyme molar ratios of 1:10. (b) Percent of maximum ThT signal at the endpoint of aggregation. Each bar represents an average of three individual experiments. (c) TEM analysis of the endpoint samples in the absence or presence of the different α_2_M variants, with scale bars representing 100 nm and numbers corresponding to the lanes in gel analysis. (d) SDS–PAGE of the endpoint supernatants shows no soluble protein in I59T lysozyme incubated alone (1), whereas soluble protein is present for samples containing native α_2_M (2), trypsin-α_2_M (3) and NH_4_Cl-activated α_2_M (4). Soluble I59T lysozyme is shown in lane S and lane M shows molecular mass markers. (e) SDS–PAGE of the solubilized endpoint pellets showing a significant quantity of protein present for I59T incubated alone (1p), and also trace quantities of protein present for samples containing native α_2_M (2p), trypsin-α_2_M (3p) and NH_4_Cl-activated α_2_M (4p).

Native α_2_M has previously been shown to inhibit the amorphous and fibrillar aggregation of a range of proteins by increasing their solubility [5,11,12,16]. To determine if activated α_2_M can also prevent amyloid formation, we compared the effect of native α_2_M and trypsin-α_2_M on the fibril formation of I59T lysozyme and the amyloidogenic peptide Aβ_1–42_. Previously reported conditions for generating trypsin-α_2_M vary greatly [2,5,20]; therefore, in this study we used an optimized method to obtain preparations of trypsin-α_2_M that were completely activated but not degraded (Supplementary Fig. 1). The aggregation behavior of I59T lysozyme is well established and this system has been used to study the effects on fibril formation of the extracellular chaperones clusterin, haptoglobin and native α_2_M [12,21]. In this study, the kinetics of aggregation show a lag phase of ca. 50 min, followed by a rapid growth phase that reaches a plateau after ca. 150 min (Fig. 1a, black line). α_2_M, present at a molar ratio of 10:1 (lysozyme-to-α_2_M), results in a dramatic decrease in thioflavin-T (ThT) fluorescence over the course of the assay (Fig. 1a; red line). When trypsin-α_2_M is incubated with I59T lysozyme the ThT fluorescence is again, significantly suppressed (Fig. 1a; blue line). At the endpoint of the fibril formation, the presence of both native α_2_M and trypsin-α_2_M results in over a 90% decrease in ThT signal relative to the I59T lysozyme sample alone (Fig. 1b).

TEM images of the ThT assay endpoint samples demonstrate that while I59T lysozyme alone forms fibrillar structures there is no evidence for such structures when I59T lysozyme is incubated under the same conditions with native α_2_M or trypsin-α_2_M (Fig. 1c). SDS–PAGE analysis of the endpoint supernatants reveals that no detectable I59T lysozyme remains in solution when incubated alone, whereas in the presence of native α_2_M, a large majority (>90%) of lysozyme remains soluble (Fig. 1d). The I59T lysozyme also remains in the soluble fraction when incubated with trypsin-α_2_M and shows no evidence of proteolytic degradation (Fig. 1d). Conversely, the pellet fractions (solubilized with 8 M urea), shows a large proportion of I59T lysozyme in the I59T alone sample (Fig. 1e, lane 1p) and only trace amounts (less than 10%) of lysozyme present in samples incubated with native α_2_M and trypsin-α_2_M (Fig. 1e, lane 2p and 3p). This finding is consistent with the fraction of the maximum ThT signal observed at the aggregation endpoints (Fig. 1b). In separate experiments, incubation of monomeric I59T lysozyme with trypsin or trypsin-α_2_M does not result in the appearance of any degraded protein in the soluble fractions after 120 min of incubation under the aggregation conditions used (Supplementary Fig. 2a), in addition, trypsin alone has no effect on I59T fibril formation (Supplementary Fig. 2b). However, it is noted that small quantities of protein fragments (less than 5% total protein) are apparent in the SDS–PAGE analysis of the pellet samples after 300 min incubation. These fragments may be the result of residual trypsin-α_2_M activity, but they appear to be aggregation prone as they are only apparent in small quantities in the insoluble pellet sample. Taken together these results reveal that, native α_2_M and trypsin-α_2_M are able to suppress I59T fibril formation predominantly via chaperone action.

**Fig. 2:**
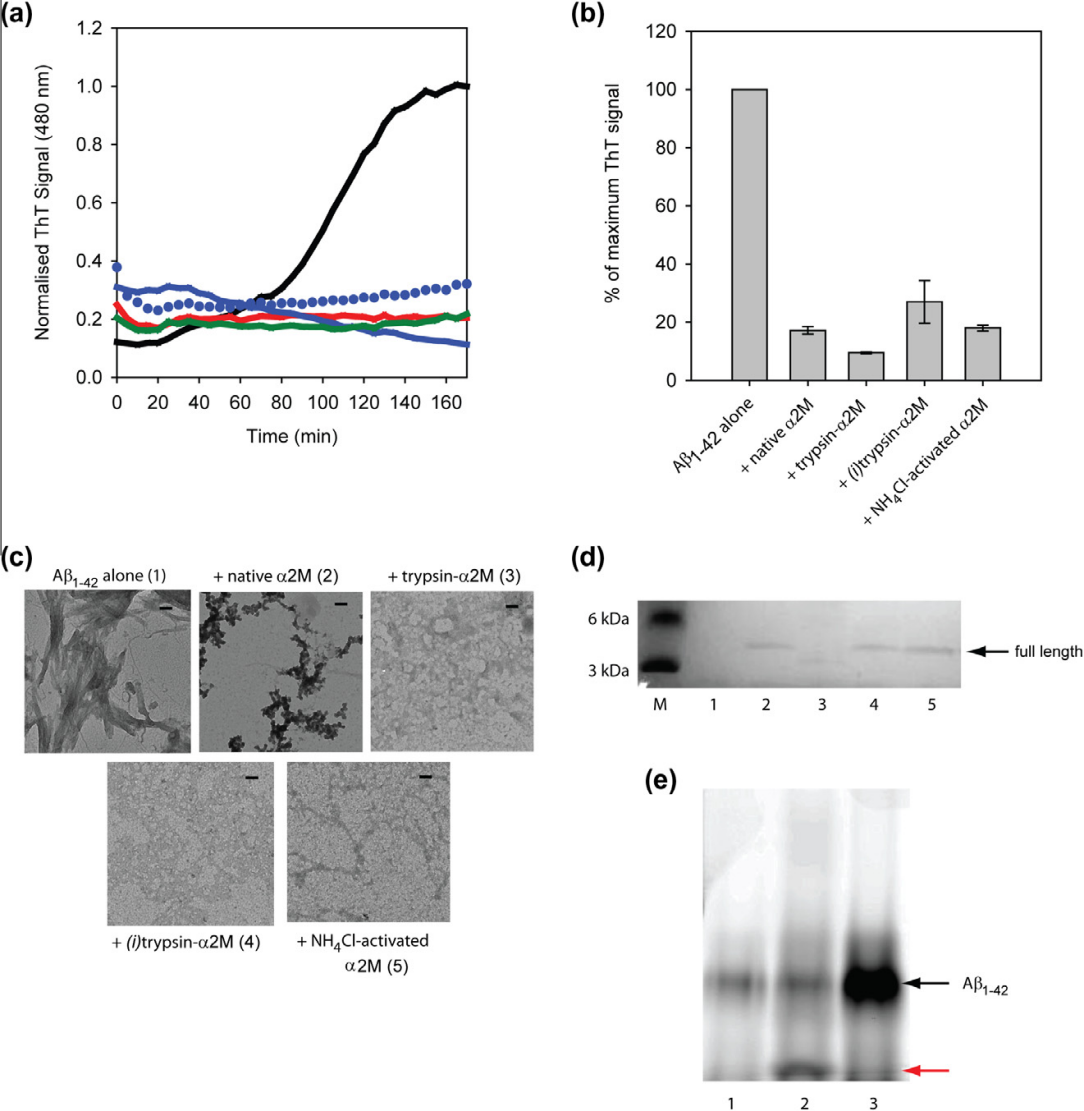
Effects of α_2_M variants on Aβ_1–42_ fibril formation. (a) In vitro fibril formation of Aβ_1–42_, incubated alone (black), with native α_2_M (red), with trypsin-α_2_M (blue), with (*i*)trypsin-α_2_M (blue circles) or with NH_4_Cl-activated α_2_M (green), using α_2_M-to-Aβ_1–42_ molar ratios of 1:10. (b) Percent of maximum ThT signal at the endpoint of aggregation. Each bar represents the average of three individual experiments. (c) TEM analysis of the endpoint samples in the absence or presence of the different α_2_M variants, with scale bars representing 100 nm and numbers corresponding to the lanes in gel analysis. (d) SDS–PAGE analysis of the endpoint supernatants shows no soluble protein for Aβ_1–42_ incubated alone (1), but soluble peptide present when incubated with native α_2_M (2). Incubation with trypsin-α_2_M (3) results in no full-length Aβ_1–42_ peptide, whereas the incubation with (*i*)trypsin-α_2_M (4) and NH_4_Cl-activated α_2_M (5) have full-length peptide present. Lane M shows molecular mass markers. (d) Fluorescence image of SDS–PAGE analysis of HiLyte-488 labelled Aβ_1–42_, alone (lane 1) and after incubation with trypsin-α_2_M (lane 2) or (*i*)trypsin-α_2_M (lane 3). The presence of trypsin-α_2_M results in an increase in Aβ_1–42_ fragments (red arrow).

We next evaluated whether trypsin-α_2_M could also suppress Aβ_1–42_ fibril formation. Under the conditions used here, aggregation of Aβ_1–42_ shows a lag of ca. 70 min, followed by a rapid growth phase and a plateau at ca. 150 min (Fig. 2a, black line). Consistent with previous studies [12], the presence of native α_2_M at a 10:1 (Aβ_1–42_-to-α_2_M) molar ratio dramatically reduces the time-dependent increase in ThT fluorescence (Fig. 1a; red line). At the same molar ratio, the presence of trypsin-α_2_M also results in a suppression of ThT fluorescence (Fig. 2a, solid blue line). This suppression in ThT signal is over 80% for both the presence of native α_2_M and trypsin-α_2_M at the endpoint of the assay (Fig. 2b). In all samples containing Aβ_1–42_ there is a small ThT fluorescence signal at the start of the assay, likely to be due to some ThT positive aggregates being present in the stock peptide solutions. This level remains constant over the time course for the samples containing native α_2_M, but decreases slightly in the presence of trypsin-α_2_M. We suspect that this may be due to the ability of trypsin-α_2_M to degrade these ThT positive species.

TEM images of the ThT assay endpoint samples show that fibrillar aggregates are formed by Aβ_1–42_ incubated alone; however, in the presence of either native α_2_M or trypsin-α_2_M, the number of well-defined fibrils is reduced and most aggregates appear to be amorphous (Fig. 2c). Analysis of the endpoint supernatants by SDS–PAGE reveals that incubation of Aβ_1–42_ with trypsin-α_2_M results in proteolysis of the peptide (Fig. 2d, lane 3). This result is consistent with previous work showing that α_2_M-trapped proteases remain active against small substrates including Aβ_1–42_ [22]. Therefore, it appears that trypsin-α_2_M prevents Aβ_1–42_ fibril formation, under these conditions at least partly via degradation of the Aβ_1–42_ peptide to form smaller species that remain soluble. Interestingly, this mechanism for inhibiting fibril formation may not be restricted to just Aβ_1–42_. We have also observed that trypsin-α_2_M can suppress the fibril formation of reduced and carboxymethylated α-lactalbumin by a process which involves proteolysis of the full-length protein (Supplementary Fig. 3).

Given that trypsin-α_2_M can degrade polypeptides which can enter the activated α_2_M cage, it is necessary to inactivate the bound trypsin to examine, in isolation, whether trypsin-α_2_M possesses chaperone activity similar to native α_2_M. Trypsin-α_2_M and trypsin-α_2_M after treatment with a small molecule protease inhibitor ((*i*)trypsin-α_2_M) migrate similarly when analyzed by native PAGE, suggesting that protease inactivation does not grossly affect the structure of the covalent complex (Supplementary Fig. 1). Incubation of fluorophore-labelled Aβ_1–42_ with trypsin-α_2_M shows that pre-treatment of the latter with protease inhibitors prevents detectable proteolysis of Aβ_1–42_ (Fig. 2e), however, the (*i*)trypsin-α_2_M retains the ability to inhibit Aβ_1–42_ aggregation (Fig. 2a, blue circles). Significantly, analysis of the endpoint supernatants reveals that soluble, full-length Aβ_1–42_ is present in the (*i*)trypsin-α_2_M sample and no degradation fragments are observed (Fig. 2d, lane 4). Analysis by TEM confirms that no fibrils are present in the Aβ_1–42_ sample containing (*i*)trypsin-α_2_M (Fig. 2c).

To confirm that chaperone activity of activated α_2_M is not reliant on the presence of the bound protease, we tested the ability of NH_4_Cl activated-α_2_M to suppress fibril formation. Data from aggregation assays show that NH_4_Cl-activated α_2_M effectively suppresses the ThT fluorescence associated with fibril formation by Aβ_1–42_ peptide or I59T lysozyme (Fig. 1a and Fig. 2a, green lines). TEM images of the endpoint samples show that only traces of fibrillar species are present in either the Aβ_1–42_ or the I59T sample containing NH_4_Cl-activated α_2_M. Furthermore, SDS–PAGE analysis of the endpoint supernatants demonstrates that the presence of NH_4_Cl-activated α_2_M increases the proportion of both client proteins remaining in their soluble, full-length forms at the endpoint of the assays (Fig. 1d, lane 4 and Fig. 2d lane 5). These results confirm that activated α_2_M can influence the solubility of polypeptides regardless of whether or not it is complexed to a protease molecule.

## 4 Discussion

In the work presented here, we show that activated α_2_M, despite a large conformational change upon activation, retains the ability to suppress fibril formation. From earlier work, it is clear that α_2_M has distinct binding sites for proteases and misfolded proteins as the binding of a misfolded client protein does not prevent protease trapping [6]. In the current study, we demonstrate that the presence of a bound protease, regardless of whether or not the protease is pharmacologically inhibited, does not significantly reduce chaperone activity of α_2_M. Moreover, α_2_M remains an active chaperone after direct activation using small molecules.

In vivo, activated α_2_M is rapidly cleared from circulation [2] and typically represents only 0.17–0.7% of the total α_2_M in blood plasma of adults [23]. The activated α_2_M plasma concentration is, however, increased in many disease states including pancreatitis, multiple sclerosis and sepsis [23–25]. Moreover, the onset of some diseases, such as periodontitis, diabetic retinopathy and inflammatory joint disease results in increased activated α_2_M levels in other extracellular fluids [26–28]. Although enhanced concentrations of activated α_2_M have been largely attributed to increased protease trapping, it has been reported that interaction with proteases only partially accounts for the total activated α_2_M present in synovial fluid [28]; higher levels of both protease-activated and amine-activated α_2_M may therefore be significant for facilitating clearance of aberrant clients via LRP. Interestingly, aggregates of Aβ_1–40_ and amylin have been shown to activate the plasmin protease system [29]. Thus it is possible that concentrations of plasmin-activated α_2_M may also be increased in response to the accumulation of misfolded proteins.

In conclusion, we provide evidence that protease-activated α_2_M has two distinct mechanisms for inhibiting amyloid formation: (i) via protease–α_2_M-mediated degradation of amyloidogenic substrates and (ii) by a chaperone action that prevents misfolded clients from continuing along the amyloid forming pathway. In the absence of proteases, activated α_2_M is able to inhibit fibril formation via the latter function only. It is tempting to speculate that the chaperone activity of protease-activated α_2_M may target misfolded proteins to the trapped protease, thereby providing a specific mechanism for degradation of amyloidogenic proteins in extracellular fluids. Clearly, further studies are required to substantiate this proposition; however, a greater understanding of the mechanisms by which α_2_M is able to prevent protein aggregation and facilitate the disposal of misfolded peptide and protein molecules could, in future, provide potential therapeutic targets for amyloidosis.
