# Supplementary material for: Protease-activated alpha-2-macroglobulin can inhibit amyloid formation via two distinct mechanisms
Source: FEBS Lett. 2013 Mar 1;587(5):398–403. doi: 10.1016/j.febslet.2013.01.020 (PMC3581772; doi:10.1016/j.febslet.2013.01.020)
Supplement: Supplementary data 1 [file mmc1.pdf]

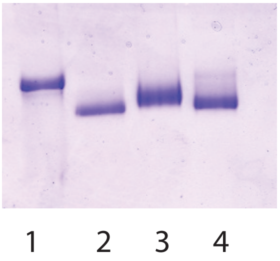

Supplementary Figure 1: Effect of activation on the migration of α_2M by native PAGE analysis

The gel shows the migration of native α_2M (lane 1), trypsin-α_2M (lane 2), *(i)*trypsin-α_2M (lane 3) and NH_4Cl-activated α_2M (lane 4) on a 3-8% Tris-acetate gel after electrophoresis at 150 V for 1.5 hr.

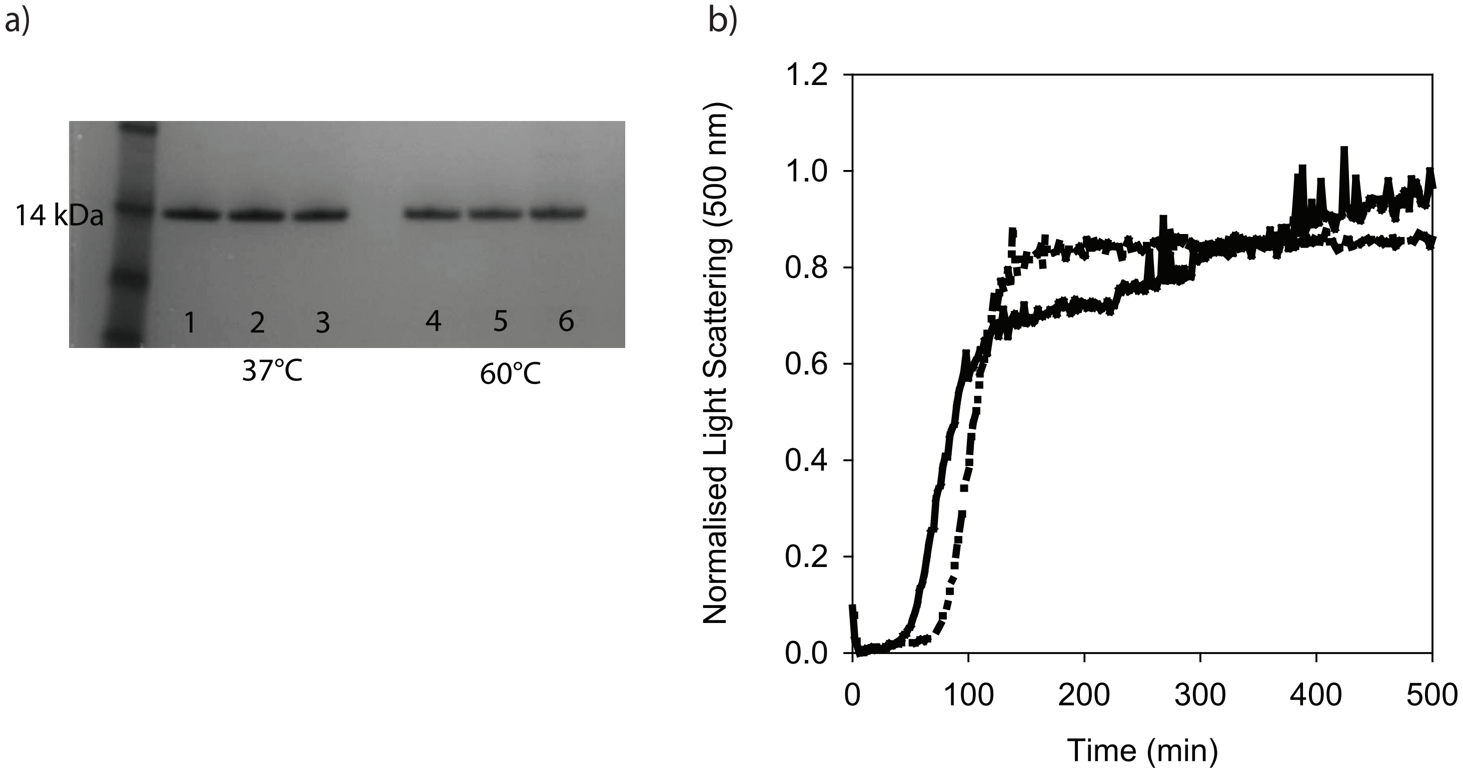

Supplementary Figure 2:
a) SDS-PAGE analysis of I59T lysozyme samples which were incubated at 37°C or 60°C for 2 hr in the absence (lanes 1 and 4) and presence of trypsin (lanes 2 and 5) or trypsin-α_2M (lanes 3 and 6). The molar ratios of trypsin-to-I59T and trypsin-α_2M-to-I59T were 1:5 and 1:10 respectively. The gel was run under reducing conditions to confirm that no degradation of full length I59T protein had occurred under either set of conditions. **b)** *In vitro* fibril formation (60°C, pH 5.0) of I59T lysozyme (solid line) in the absence or presence of trypsin (dashed line) at a 1:10 (trypsin-to-lysozyme) molar ratio as monitored by light scattering. The presence of trypsin does not significantly affect the overall fibril formation of I59T lysozyme.

a)

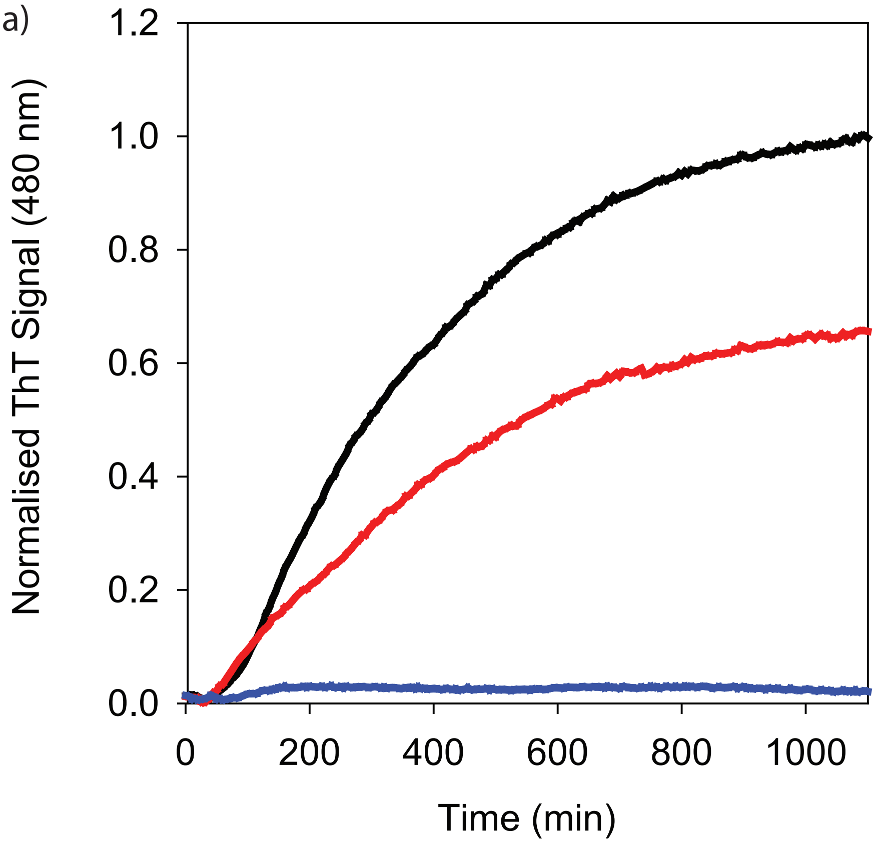

b)

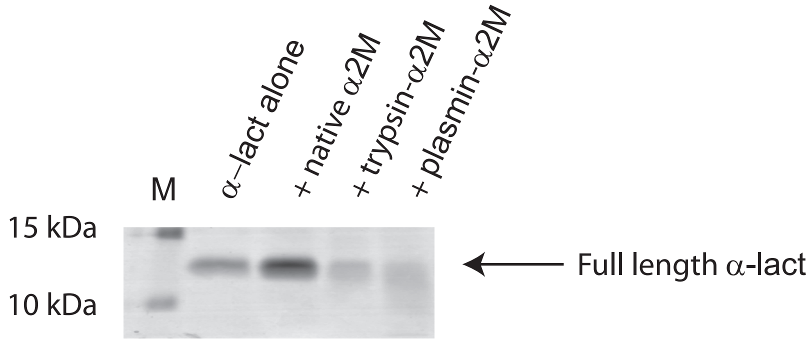

Supplementary Figure 3: Effects of native and trypsin- α$_2$M on fibril formation by α-lactalbumin

a) *In vitro* fibril formation (37°C, pH 7.4) of α-lactalbumin (α-lact) alone (black) or in the presence of native α$_2$M (red) or trypsin-α$_2$M (blue) as monitored by ThT fluorescence. The molar ratio of α $_2$M-to-α-lact used was 1:10. The experiment was performed in triplicate. **b)** SDS-PAGE analysis of the endpoint samples. Some soluble protein remains in the sample containing α-lact alone, but more full-length, soluble α-lact is present in the sample containing native α$_2$M. After co-incubation with trypsin α$_2$M, SDS-PAGE analysis shows less full-length α-lact than does a corresponding sample of α-lact after co-incubation with plasmin-α$_2$M.
